# Neutrophil kinetics of *Shigella* infection in *Macaca mulatta* (Rhesus macaques)

**DOI:** 10.3389/fcimb.2026.1810976

**Published:** 2026-07-01

**Authors:** Ziyuan He, Brandon J. Beddingfield, Harriet Hammond, Elizabeth S. Didier, Lucy Freytag, Marcelo J. Kuroda, Chad J. Roy

**Affiliations:** 1 Division of Immunology, Tulane National Biomedical Research Center, Covington, LA, United States; 2 Department of Microbiology and Immunology, Tulane School of Medicine, New Orleans, LA, United States; 3 Division of Microbiology, Tulane National Biomedical Research Center, Covington, LA, United States; 4 Center for Airborne Infection & Transmission Science, Tulane University School of Medicine, New Orleans, LA, United States; 5 Department of Medicine, Tulane University School of Medicine, New Orleans, LA, United States

**Keywords:** gastroinmtestinal diseases, model development, neutrophil, nonhuman primate, *Shigella*

## Abstract

**Introduction:**

Neutrophil migration is a hallmark of acute inflammation and represents a key component of the early innate immune response to bacterial infection. We previously demonstrated the ability to track neutrophil movement from bone marrow to blood and subsequently to tissues using 5-bromo-2’-deoxyuridine (BrdU) pulse labeling in healthy adult rhesus macaques (*Macaca mulatta*), observing a consistent pattern of neutrophil kinetics during homeostasis.

**Methods:**

In this study, we extend our analyses to investigate the kinetics of neutrophil movement during acute inflammatory responses using *Shigella sonnei*, the causative agent of bacillary dysentery, as a model for acute bacterial infection. Twelve adult rhesus macaques were divided into three groups and challenged with varying doses of *S. sonnei* via gastric-oral lavage.

**Results:**

BrdU labeling revealed significant neutrophil consumption from blood into tissues and replenishment from bone marrow to blood as early as three days post-challenge, with a transient increase in neutrophil counts in blood at day nine post-challenge. Moreover, the extent of neutrophil kinetic changes correlated with the doses of *S. sonnei*.

**Discussion:**

These findings suggest that BrdU-labeled neutrophil kinetics provide valuable insights into neutrophil dynamics *in vivo*. This knowledge can be crucial for future studies that monitor acute inflammatory responses to infectious agents using nonhuman primate models. Additionally, it may aid in understanding the mechanisms of disease development and in creating effective intervention strategies.

## Introduction

Neutrophils, the most abundant leukocytes in human circulation, are traditionally seen as key players in acute inflammatory responses. Due to their vital immune functions and short lifespan, they require continuous production in the bone marrow, a process known as granulopoiesis (Soehnlein et al., 2017). Neutrophil count is a critical hematological parameter for assessing acute inflammation, with both neutropenia and neutrophilia indicating various inflammatory conditions (Honda et al., 2016). However, interpreting absolute neutrophil counts (ANC) is complex due to the dynamic balance between neutrophil production in the bone marrow, migration between circulating and marginal pools, and clearance or apoptosis in tissues. Consequently, neutrophil counts often fluctuate, complicating clinical interpretations and underscoring the need for a deeper understanding of neutrophil roles and kinetics during acute and chronic inflammation (Margraf et al., 2022). Studying neutrophil kinetics in humans presents significant challenges due to both ethical and practical constraints. Nonhuman primates (NHPs), like rhesus macaques, share close genetic and physiological similarities with humans, making them invaluable models for researching human diseases and immune system functions.

*Shigella* spp. are Gram-negative intracellular bacteria that are transmitted via the fecal-oral route, causing bacillary dysentery in both humans and NHPs. *Shigella* represents a significant global health concern, particularly affecting young children under the age of five. Among the four species – *S. sonnei*, *S. boydii*, *S. dysenteriae*, and *S. flexneri* - *S. flexneri* and *S. sonnei* are the primary species responsible for endemic shigellosis, with *S. flexneri* being the most common species isolated from captive NHPs (Maxwell et al., 2022). Symptoms range from severe, life-threatening dysentery to asymptomatic carrier states (Maxwell et al., 2022). NHPs, such as rhesus macaques, are the only animal models that closely replicate *Shigella* infection as seen in human disease. When orally infected with *S. flexneri*, these primates develop symptoms of diarrhea and intestinal damage (Alphonse and Odendall, 2023).

Neutrophils play a crucial role in both host immunity and the pathogenesis of *Shigella* infection. In humans and rhesus macaques, shigellosis is characterized by significant neutrophil infiltration in the gut, which destabilizes gut integrity (Philpott et al., 2000; Islam et al., 2016). Upon entry through microfold cells into the gut submucosa, *Shigella* bacteria are phagocytosed by macrophages, which subsequently undergo apoptosis and release IL-1β to induce inflammation. Infected epithelial cells secrete IL-8, attracting neutrophils to the local tissues (Philpott et al., 2000). Emergency granulopoiesis, marked by increased bone marrow hematopoietic production to compensate for neutrophil depletion due to microbial infections, has been observed in animal models and human clinical studies (Manz and Boettcher, 2014). In a zebrafish larvae *Shigella* infection model, granulopoiesis plays critical protective roles (Willis et al., 2018). However, quantifying granulopoiesis remains challenging, with ANC used as a proximate estimation (Harrold et al., 2020).

5-bromo-2’-deoxyuridine (BrdU), a thymidine analog, incorporates into hematopoietic progenitor cells in the bone marrow, serving as a tool to characterize myeloid lineage cell differentiation *in vivo*. In earlier studies, we demonstrated that *in vivo* BrdU pulse-chase experiments could monitor changes in blood monocyte turnover rates, identify newly divided neutrophils in the bone marrow, and track their presence in blood circulation and tissues during homeostasis, aging, and viral and bacterial infections in rhesus macaques (Hasegawa et al., 2009; Mehra et al., 2012; Sugimoto et al., 2015; He et al., 2018; He et al., 2021). We observed that neutrophil kinetics were consistently and tightly regulated during homeostasis in adult rhesus macaques (He et al., 2018).

In the present study, we investigated neutrophil kinetics using *in vivo* BrdU pulse-chase labeling to better understand the host response and neutrophil movement during acute bacterial exposure, using *Shigella* infection in rhesus macaques as an experimental model. Rhesus macaques frequently harbor circulating antibodies to *S. flexneri*, as they are natural carriers (Sestak et al., 2003). Therefore, to avoid a cross-reactive immune response from any naturally acquired immunity to previous *S. flexneri* exposure or infection, *S. sonnei* was used as the infecting bacterial species. In this disease modeling effort, gastric-oral gavage exposure of *S. sonnei* was performed in rhesus macaques to induce clinical shigellosis. To further elucidate the host response, in addition to clinical signs (e.g., diarrhea), peripheral neutrophil counts and corresponding kinetics were defined using timed administration of BrdU.

## Materials and methods

### Rhesus macaques

Twelve Indian-origin young adult rhesus macaques (2 females and 10 males) aged between 6.9 to 15.3 years from the Tulane National Biomedical Research Center were used to study neutrophil kinetics following *Shigella* inoculation (**Supplementary Table 1**). The animals were specific pathogen-free of SIV, Simian Betaretrovirus (formerly known as Type D Simian Retrovirus), Macacine herpesvirus 1 (i.e. herpes B virus), and Simian T-cell Leukemia Virus type 1 (STLV-1). Since *Shigella* occurs naturally in primates, a pool of 60 animals was initially prescreened for existing *S. flexneri* and *S. sonnei* LPS serum IgG antibodies. An arbitrary 1:40 endpoint cutoff was used as inclusion criteria for this study. These animals were in excellent health and their stool cultures were negative for *Shigella*. All procedures were performed in accordance with the National Institutes of Health Guide for the Care and Use of Laboratory Animals and approved by the Tulane University Institutional Animal Care and Use Committee (National Research Council (US) Committee for the Update of the Guide for the Care and Use of Laboratory Animals, 2011).

### *Shigella* challenge

**Figure 1:**
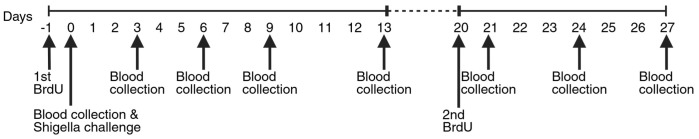
Schematics of *Shigella* challenge studies. Twelve young adult rhesus macaques (n = 4 per group) were administered BrdU (60 mg/kg body weight) intravenously on day -1, followed by an oral challenge with a single dose of different concentrations of *S. sonnei* on day 0 (Group 1: 10^11^ CFU; Group 2: 2×10^10^ CFU; Group 3: 1.5×10^10^ CFU). Whole blood samples were collected on days 0 (pre-challenge), 3, 6, 9, and 13 (days 1, 4, 7, 10, and 14 post-BrdU injection), stained, and analyzed for immunophenotyping and BrdU incorporation. On day 20, a second BrdU injection (60 mg/kg body weight) was administered intravenously, and blood samples were collected on days 21, 24, and 26 (days 1, 4, and 7 post-BrdU injection) for immunophenotyping and BrdU incorporation analysis. CFU, colony forming unit.

The *S. sonnei* strain WRAIR I Virulent (BEI NR-519) was used for all challenges within this study. Prior to *S. sonnei* inoculation, animals were fasted for ~16 hours and stomach acidity were neutralized with sodium bicarbonate administered by gastric-oral gavage. An inoculum of cultured *S. sonnei* organisms was delivered intragastrically in 20 mL of sterile saline at different doses into three groups of rhesus macaques (day 0; n = 4 for each group: Group 1 received 1 ×10^11^ CFU, Group 2 received 2×10^10^ CFU, and Group 3 received 1.5×10^10^ CFU) (**Figure 1**). These inoculum doses were chosen as to align with prior *Shigella* studies in nonhuman primates (Labrec et al., 1964; Shipley et al., 2010). *Shigella*- induced diarrhea was quantified using a modified Bristol scale, in accordance with the following parameters: daily stool collections were analyzed for the presence of occult blood and cultured in Hektoen, MacConkey, and TSI agar to determine shedding of *Shigella* (**Supplementary Table 2**). We attempted to control for age-related changes in neutrophil kinetics by creating groups with a mixture of ages.

### BrdU administration and blood collection

For neutrophil kinetics studies, the thymidine analogue BrdU (catalog number B5002-100G; Sigma-Aldrich, St. Louis, MO,USA) was prepared at 30 mg/ml in endotoxin-free PBS (catalog number TMS-012-A; EMD Millipore, Burlington, MA, USA), filter sterilized through a 0.2 µm polyethersulfone membrane (Steriflip, catalog number SCGP00525 or 09-740-2A; ThermoFisher Scientific, Waltham, MA, USA), and administered intravenously at a dose of 60 mg/kg body weight into each rhesus macaque on day -1 pre-*Shigella* challenge. Blood samples were collected with EDTA anticoagulant for BrdU staining, flow cytometry, and hematology analyses on days 1, 4, 7, 10, and 14 after initial BrdU administration (**Figure 1**).

### Flow cytometry and hematology analyses

Cellular immunophenotyping and staining for BrdU incorporation were performed as previously described (Sugimoto et al., 2015; He et al., 2018; He et al., 2021). Briefly, 200 µl of EDTA–anticoagulated whole blood were washed with PBS and stained with surface monoclonal antibodies (**Supplementary Table 3**). Red blood cells were lysed with FACS lysing solution (catalog number 349202; BD Biosciences, San Jose, CA, USA), and remaining cells were permeabilized using a three-step Cytofix/Cytoperm protocol per the manufacturer’s instructions (BD Biosciences). For analysis of BrdU incorporation, cells were incubated with DNase I (catalog number DN25, Sigma-Aldrich) at 37°for 1 hour and then stained with anti-BrdU antibody for 20 minutes at room temperature. After washing, cells were fixed in 250 µL of 1% paraformaldehyde in PBS. Samples were acquired with a LSRFortessa flow cytometer (BD Biosciences) and data were analyzed with FlowJo software (version 10; FlowJo, LLC, Ashland, OR, USA). Hematology analyses were performed on a Sysmex XT-2000iV automated hematology analyzer (Sysmex America, Lincolnshire, IL). Absolute cell counts for neutrophils were directly measured from the automated hematology analyzer. Gating for neutrophils was performed as previously demonstrated (He et al., 2018). Briefly, granulocytes were first gated from intermediate to high forward scatter (FSC) and side scatter (SSC) fractions and then separated based on the expression of HLA-DR, with neutrophils negative for CD123 (FSC/SSC^high/dim^, HLA-DR^−^, CD3^−^, CD20^−^, CD123^−^). Neutrophil content was confirmed via myeloperoxidase expression.

### Statistical analyses

The nonparamtric Friedman test and post-analysis comparisons were performed on neutrophil counts at different days pre- and post-*Shigella* challenge. Kruskal-Wallis analyses were used to compare results between different dosage groups. We applied a previously established neutrophil kinetic model, derived from healthy adult macaques (He et al., 2018), to fit the kinetics following *Shigella* challenge and estimated the time at which BrdU-labeled neutrophils peak in circulation. Graphs were prepared using GraphPad Prism 7.0 (GraphPad Software, San Diego, CA, USA). A *p* value <0.05 was considered statistically significant.

## Results

### Neutrophil kinetics shift in response to acute *Shigella* infection

**Figure 2:**
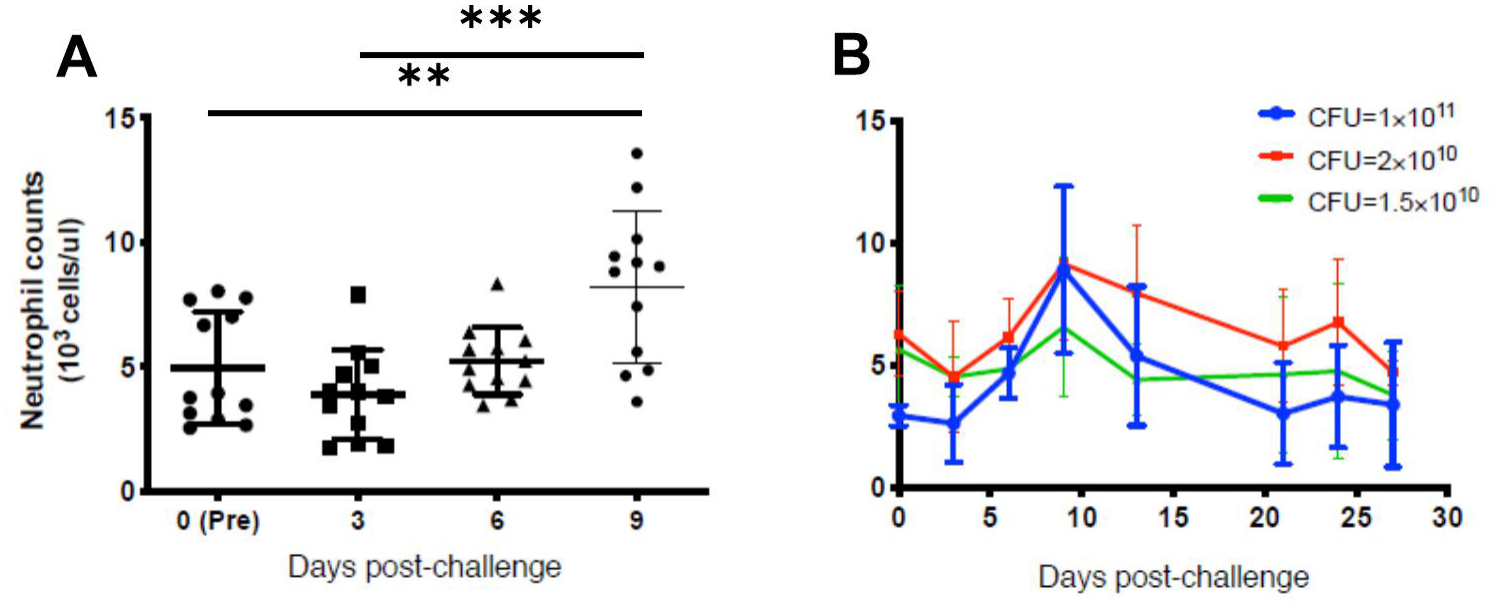
Reversible neutrophilia is observed during acute *Shigella* challenge. Neutrophil counts were measured at pre-challenge (day 0) and post-*Shigella* challenge in all three groups of rhesus macaques. **(A)** The Friedman test and *post-hoc* comparisons were performed among neutrophil counts at different days pre- and post- challenge (**p < 0.01, ****p < 0.0001). **(B)** Means ± SD of neutrophil counts during *Shigella* challenge for each dosage group (n = 4). CFU, colony forming unit.

To investigate the effects of acute *Shigella* infection on neutrophil kinetics, we monitored neutrophil numbers using complete blood cell counts and assessed kinetics by immunophenotyping and flow cytometry staining of BrdU-labeled neutrophils. We administered BrdU (60 mg/kg body weight) intravenously to 12 young adult rhesus macaques (n = 4 per group) on day -1, and then challenged them with a single dose of different concentrations of *S. sonnei* (Group 1: 1 ×10^11^ CFU; Group 2: 2×10^10^ CFU; Group 3: 1.5×10^10^ CFU) orally on day 0 (**Figure 1**). Animals exposed at all dose levels developed clinical shigellosis, with diarrheal indexes exceeding 3.0 (**Supplementary Table 2**). Animals tested positive for *S. sonnei* in fecal cultures 2 days post-infection and remained positive for at least 7 days post-infection, with those in the lower dose group (1.5×10^10^ CFU) resolving quicker than those in the highest dose group (1 ×10^11^ CFU) (**Supplementary Table 2**). Whole blood samples were collected on days 0 (pre-challenge), 3, 6, 9, and 13 (days 1, 4, 7, 10, and 14 after BrdU administration), stained, and analyzed for immunophenotyping and BrdU incorporation. On day 20, a second injection of BrdU (60 mg/kg body weight) was administered intravenously, and blood samples were collected on days 21, 24, and 26 (days 1, 4, and 7 after second BrdU administration) for immunophenotyping and BrdU incorporation analysis (**Figure 1**). Neutrophil counts decreased slightly but not significantly at day 3 and recovered to pre-challenge levels by day 6 post-inoculation. Interestingly, on day 9 post-inoculation, neutrophil counts were significantly higher than pre-challenge and day 3 post-challenge (**Figure 2A**). In all three *Shigella*-exposed groups, transient neutrophilia occurred on day 9 post-inoculation and quickly resolved, reaching normal neutrophil ranges by day 13 (**Figure 2B**).

**Figure 3:**
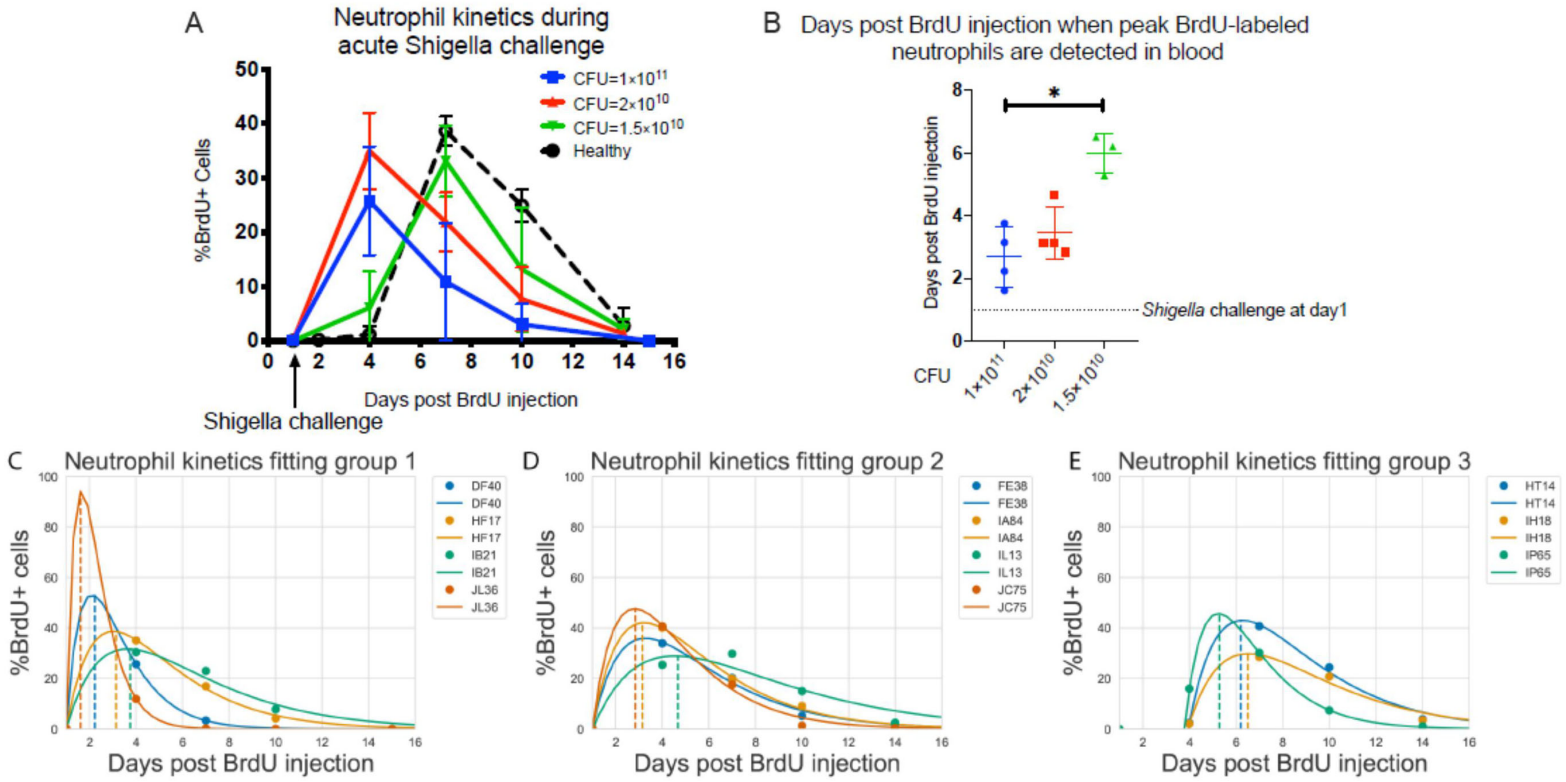
Neutrophil kinetics shift post-*Shigella* challenge. Four rhesus macaques per group received a single bolus of BrdU (60 mg/kg body weight) intravenously, and EDTA-treated blood samples were collected on various days for immunostaining and flow cytometry analyses. BrdU+ neutrophils were plotted as percentages. Doses of *S. sonnei* were inoculated 1 day post-BrdU injection. Whole blood samples were collected on days 1 (pre-challenge), 4, 7, 10, and 14 post-BrdU injection (days 0, 3, 6, 9, and 13 post-challenge). **(A)** BrdU+ cells were gated within the neutrophil population, and the mean ± SD of the percentages of BrdU+ cells for each group (n = 4) were plotted versus days post-BrdU injection. Neutrophil kinetics from healthy adult rhesus macaques (n = 11) are presented for comparison. **(B–E)** Mathematical modeling of neutrophil kinetics in all *Shigella* dosage groups. **(B)** Time post-BrdU injection for each animal when the peak BrdU-labeled neutrophils were predicted in the model in blood. Kruskal–Wallis test and multiple comparison used for analysis among the three dosage groups; p < 0.05 was considered significant, denoted by *. **(C–E)** Best-fit neutrophil kinetics for each animal in the three groups as predicted by the model. Plotted colored circles indicate actual data points measured at indicated days post-BrdU injection. Colored lines indicate the best-fit curves from the model. The x (abscissa) values of dashed lines indicate the time at peak BrdU-labeled peripheral neutrophils predicted in blood for each animal shown in **(B)**. **(C)** Group 1: 10^11^ CFU. **(D)** Group 2: 2×10^10^ CFU. **(E)** Group 3: 1.5×10^10^ CFU. CFU, colony forming unit.

BrdU was administered a day before *S. sonnei* inoculation to label the bone marrow dividing neutrophil precursors under undisturbed conditions and to investigate the movement of neutrophils *in vivo* after *S. sonnei* inoculation. On day 4 post-BrdU injection (3 days post-infection), we observed significantly higher percentages of BrdU-labeled neutrophils in blood circulation in all 12 *Shigella*-inoculated animals compared to uninfected healthy macaques, suggesting that newly divided neutrophils mobilized from the bone marrow into circulation in response to *Shigella* inoculation in the early stages of the disease (**Figure 3A**). Neutrophil kinetics were modeled as previously described, and the time post-BrdU injection was calculated for each animal to reach peak BrdU-labeled neutrophil levels in blood (dashed lines, **Figures 3C–E**) (Hasegawa et al., 2009). Results indicate that the timing of peak BrdU-labeled neutrophils is highly dependent on the *Shigella* inoculating dose; neutrophils from the highest *Shigella* inoculation dose group were released to blood stream the earliest (**Figure 3B**).

**Figure 4:**
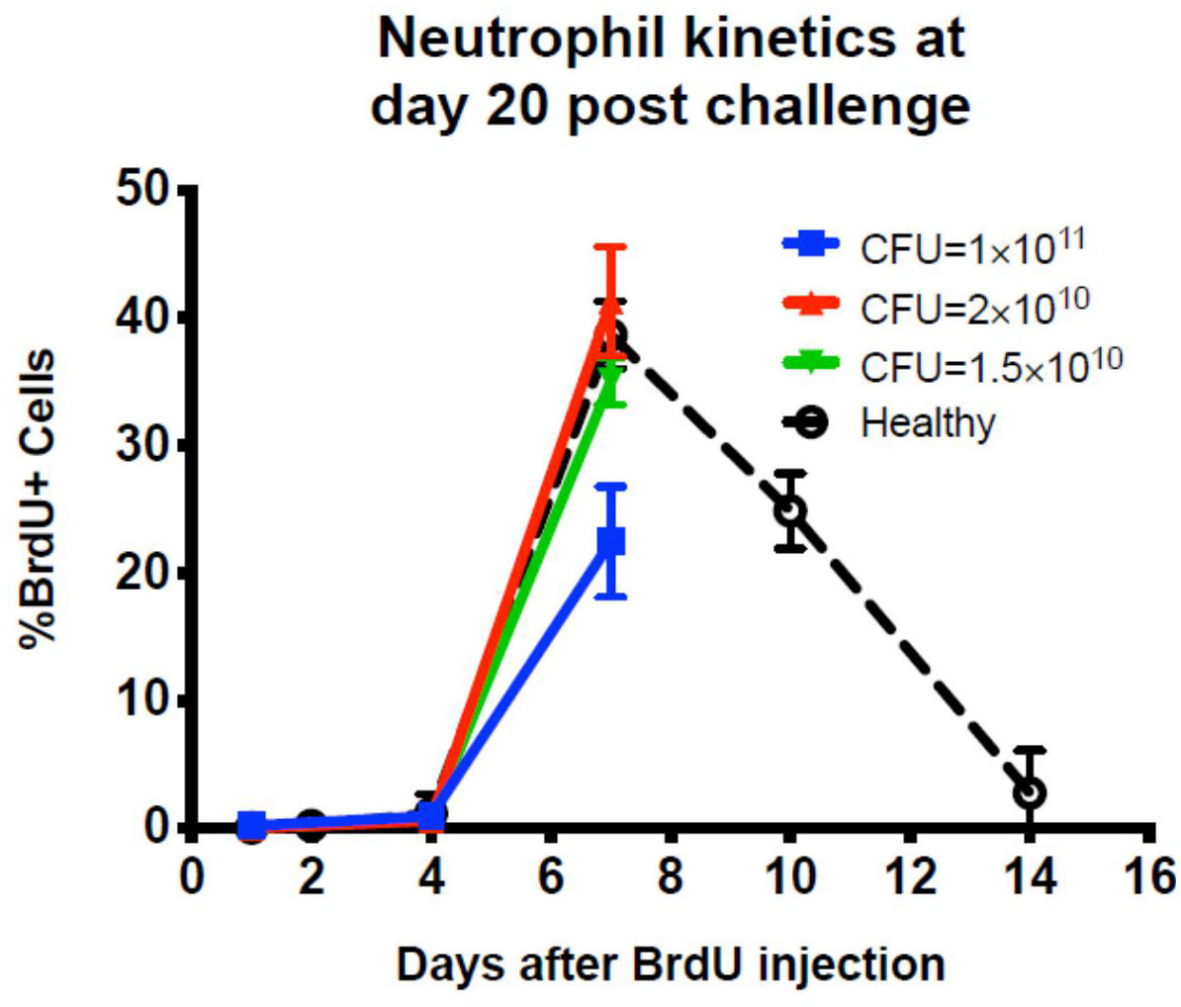
Neutrophil kinetics return to baseline when *Shigella* infection resolved. A second dose of BrdU (60 mg/kg body weight) was administered on day 20 post-challenge to measure neutrophil kinetics during disease resolution. Whole blood samples were collected on days 1, 4, and 7 post-BrdU injection (days 21, 24, and 27 post-infection) and analyzed for BrdU incorporation by immunostaining and flow cytometry. Mean values (% ± SD) of BrdU+ cells for each group (n = 4) were plotted versus days post-BrdU injection. Neutrophil kinetics from healthy adult rhesus macaques (n = 11) are presented (black dashed line) for comparison. CFU, colony forming unit.

Because BrdU-labeled neutrophils were cleared from the system by about two weeks, BrdU was administered again 20 days after the initial bacterial inoculation to monitor neutrophil kinetics in the infection resolution phase, during which fecal cultures for all animals were negative for *Shigella* (**Supplementary Table 2**). Neutrophil kinetics had reestablished to homeostatic levels in all three groups of animals, characterized by low BrdU-labeled neutrophil percentages on day 4 and higher BrdU-labeled neutrophil percentages on day 7 following the second BrdU administration, as also observed in healthy uninoculated animals (**Figure 4**).

## Discussion

Neutrophils are the primary myeloid and white blood cell (WBC) component in blood of humans and nonhuman primates (NHPs). These short-lived, first-responder immune cells patrol the body and rapidly respond to stimuli, infections, or tissue damage by eliminating pathogens and mediating acute inflammation, actions which necessitate constant replenishment from the bone marrow. Neutrophil infiltration, characterized by their movement into effector sites, is a hallmark of acute inflammation. Our research, along with others, has demonstrated that the bone marrow continuously produces large quantities of neutrophils for release into the bloodstream throughout an individual’s lifespan (Price et al., 1996; He et al., 2018; He et al., 2021). Although neutrophil counts are commonly used as a clinical parameter, the *in vivo* movement of neutrophils during acute infections is not always accurately reflected by blood cell counts. Determining the lifespan of myeloid cells in humans is challenging due to the potential toxicity of *in vivo* labeling agents and the difficulties associated with performing repeated bone marrow and blood sampling required to confirm cell kinetics. Therefore, NHPs are valuable models for *in vivo* cell proliferation labeling due to their genetic and physiological similarities to humans, as well as their comparable WBC composition (Sugimoto et al., 2015; Didier et al., 2016). Consequently, NHPs serve as important animal models for gaining a better understanding of the human immune system.

In this study, we utilized *in vivo* labeling with the thymidine analog BrdU to examine neutrophil development and kinetics in rhesus macaques during acute inflammation induced by *S. sonnei* infection. When administered, BrdU is incorporated as a thymidine analog by dividing hematopoietic stem cells in the bone marrow during DNA synthesis in the S-phase of the cell cycle, making it a reliable marker for identifying dividing cells. *Shigella* spp. are frequently encountered as enteric pathogens in captive NHPs (Sestak et al., 2003). However, the response of neutrophils to a self-resolving *S. sonnei* infection in rhesus macaques during the acute phase remained undefined. Accordingly, the kinetics of BrdU incorporation into neutrophils was tracked using the sampling strategy outlined in **Figure 1**. *Shigella* infection represents a local bacterial infection where neutrophils quickly mobilize to the local tissue, presumably the gut, to help elicit the host response to the infection. Relatively high doses of *S. sonnei* were selected as NHPs are generally resistant to clinical infection with *Shigella* spp., in contrast to humans, where the infectious dose is estimated to be as low as 10–100 organisms (DuPont et al., 1989). These data rely on the common assumption that pre-infection bone marrow proliferation are comparable across our groups, with an expected expansion and increase in turnover after bacterial challenge. Our findings document the development of neutrophils as they migrate from the bone marrow to the blood and subsequently transition into tissues in response to *Shigella* infection, though we did not directly measure the tissue migration.

We observed a transient neutrophilia at 9 days post-*S. sonnei* challenge in all three groups of rhesus macaques (**Figure 2A**). However, the kinetics, measured by the percentage of BrdU-labeled neutrophils in circulation, indicated significant movement to tissues and replenishment from the bone marrow reserve as early as 3 days post-challenge (**Figure 3A**). The neutrophilia observed at day nine post-*S. sonnei* challenge reflected compensatory neutrophil production in the bone marrow, while the actual neutrophil response to the infection occurred earlier (day three), as evidenced by the mobilization of neutrophils from the bone marrow reservoir (**Figure 3B**).

Our previous research demonstrated that in healthy rhesus macaques, BrdU-labeled neutrophils remain in the bone marrow for 4–5 days before being released and are cleared from the blood approximately 14 days after BrdU administration (He et al., 2018). Since BrdU was injected before the *Shigella* inoculation and cleared from the system quickly, and because neutrophil kinetics are consistent among young adult rhesus macaques during homeostasis, we assumed that neutrophil proliferation in the bone marrow at day -1 before inoculation, as reflected by peaks of BrdU-labeled neutrophils in the blood later on, was similar across all groups. This assumption allowed us to estimate the transit time for BrdU-labeled neutrophils to reach their peak levels in the blood in each group of animals (**Figures 3B–E**). Interestingly, the magnitude of the kinetics shift and the earlier release of neutrophils from the bone marrow directly correlated with *S. sonnei* inoculating dose, indicating that the movement of neutrophils reflected the intensity of the host response and the consumption of blood neutrophils post-challenge, possibly in a dose-dependent manner (**Figures 3C–E**). These alterations in neutrophil kinetics quickly returned to the baseline in the animals once the *Shigella* infections resolved, as indicated by negative fecal cultures (**Figure 4**; **Supplementary Table 2**).

In summary, our study demonstrates that neutrophil kinetics, measured by BrdU pulse-chase labeling, provide a valuable tool for monitoring neutrophil movement *in vivo* in rhesus macaques. During homeostasis, neutrophil kinetics are highly regulated, with significant bone marrow production observed (He et al., 2018). In response to acute infection, neutrophils are released earlier from the bone marrow reserve into the circulation, reflecting the intensity of the host response and the bacterial burden (**Figures 3A, B**). This movement, which correlates with inoculation doses, can be sensitively measured by BrdU kinetics and may serve as a useful metric for challenge model and any subsequent vaccine or therapeutic efficacy studies. Our findings also show that neutrophil kinetics return to baseline once *Shigella* infection resolves (**Figure 4**). These results emphasize the utility of NHP models in studying the kinetics of myeloid lineage cells and the pathogenesis of infectious diseases in humans. In addition, we demonstrate that kinetic shifts within neutrophils in response to infection are not fully encapsulated by purely measuring neutrophil counts. Consequently, *in vivo* BrdU pulse-chase studies are invaluable for understanding the development, kinetics, and turnover of replicating immune cells, enhancing our knowledge of innate immune responses to infectious pathogens and the role of myeloid cells in infections and inflammation. Although we focused purely upon the neutrophil response, other innate immune mechanisms may be modulated by, or important in the control of, *Shigella* infection. Future studies should focus on understanding of other responses as well.

## Data Availability

The raw data supporting the conclusions of this article will be made available by the authors, without undue reservation.

## Supplementary material

Supplementary Table 1 Demographics of Rhesus macaques used in the studies.

Supplementary Table 2 Characterization of *Shigella*-induced diarrhea and shedding.

Supplementary Table 3 List of commercial antibodies used for flow cytometry.
