# Supplementary figures and images for: Neutrophil kinetics of Shigella infection in Macaca mulatta (Rhesus macaques)
Source: Front Cell Infect Microbiol. 2026 Jul 1;16:1810976. doi: 10.3389/fcimb.2026.1810976 (PMC13369012; doi:10.3389/fcimb.2026.1810976)

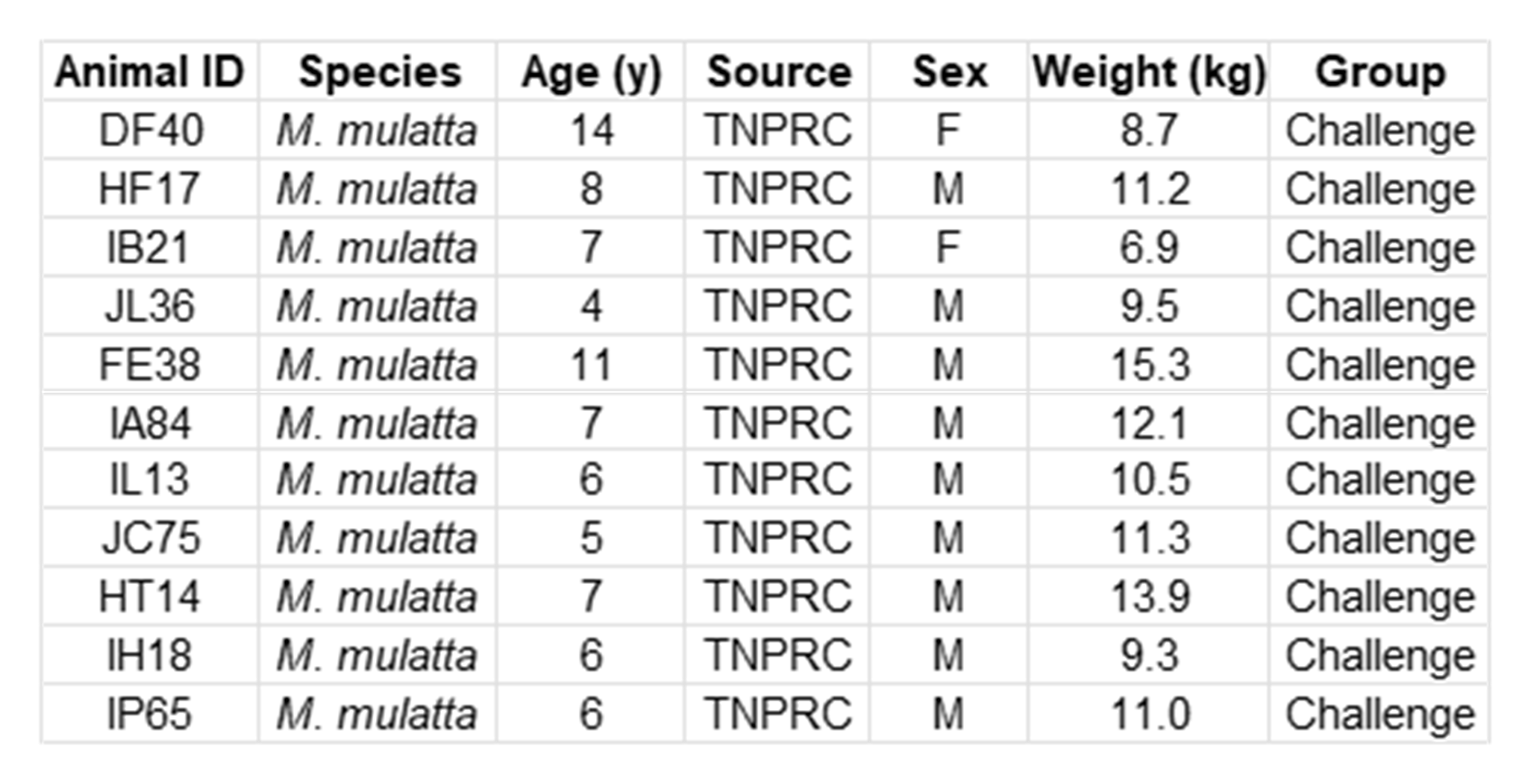

Supplement: Supplementary Table 1 — Demographics of Rhesus macaques used in the studies. [file Image1.tif]

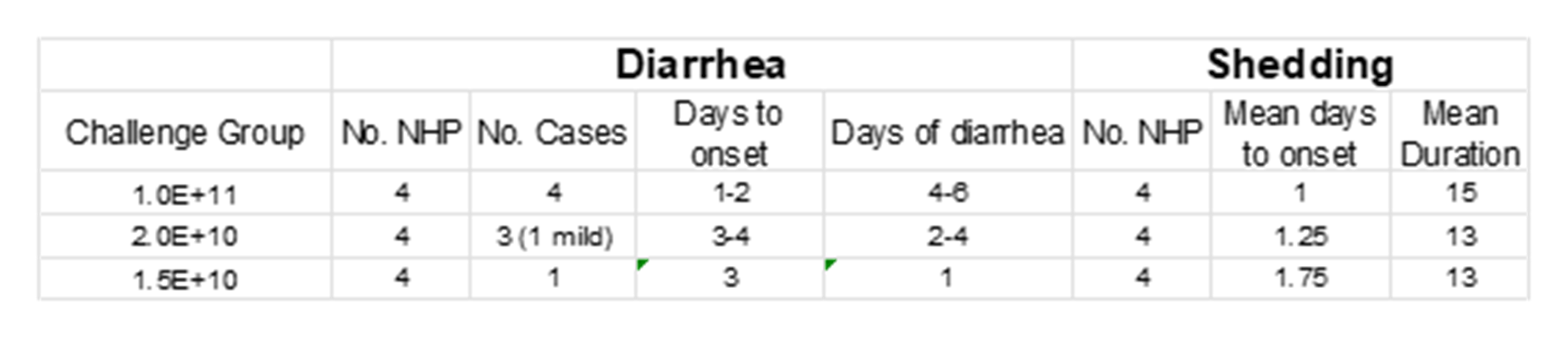

Supplement: Supplementary Table 2 — Characterization of Shigella-induced diarrhea and shedding. [file Image2.tif]

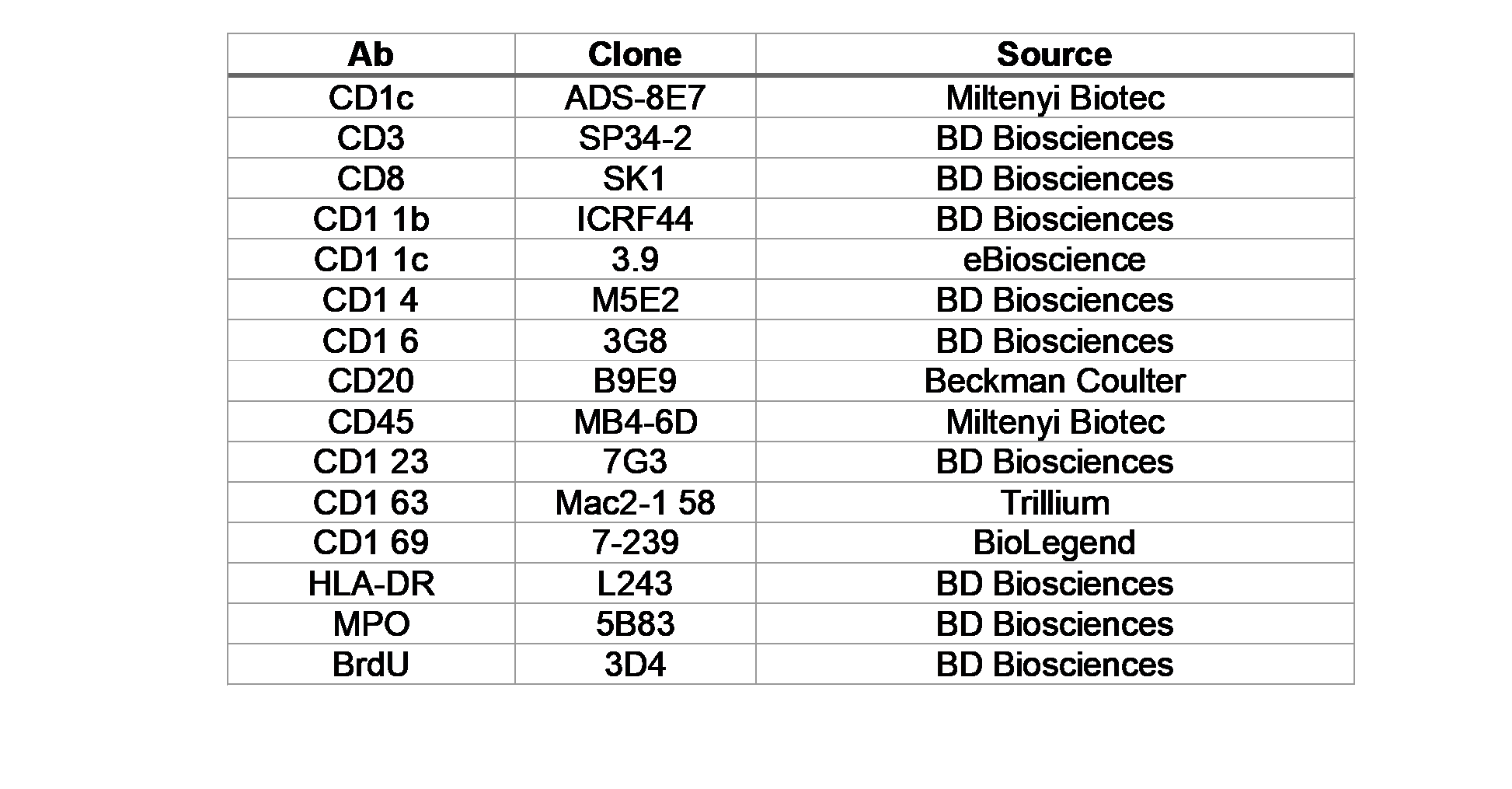

Supplement: Supplementary Table 3 — List of commercial antibodies used for flow cytometry. [file Image3.tif]
